# A case of mistaken identity: classic Kaposi sarcoma misdiagnosed as a diabetic foot ulcer in an atypical patient

**DOI:** 10.1186/s40842-019-0083-x

**Published:** 2019-07-08

**Authors:** Garneisha M. Torrence, James S. Wrobel

**Affiliations:** Michigan Medicine, Department of Internal Medicine, Division of Metabolism, Endocrinology, and Diabetes, Domino’s Farms, University of Michigan Hospital and Health Systems, (Lobby C, Suite 1300) 24 Frank Lloyd Wright Drive, Ann Arbor, MI 48106 USA

**Keywords:** Kaposi sarcoma, Malignancy, Diabetes mellitus, Foot tumor, Biopsy

## Abstract

**Background:**

The presentation of Kaposi sarcoma is divided into four known clinical subtypes. In this case report we describe classic Kaposi sarcoma in an African-American heterosexual, diabetic, seronegative human immunodeficiency virus male. Classic Kaposi sarcoma is rare in this patient demographic and can be easily misdiagnosed.

**Case presentation:**

The patient presented with a lesion between the fourth and fifth digits of his right foot which was initially diagnosed as a diabetic foot ulcer. Despite local wound care, the lesion did not resolve. A shave biopsy was performed and histopathology findings were consistent with classic Kaposi sarcoma.

**Conclusions:**

The patient tolerated local radiotherapy well and had complete resolution of his pedal lesion. There have been emerging associations between diabetes and Kaposi sarcoma. As such, clinicians should have a low threshold when considering the biopsy of suspicious pedal lesions in patients with diabetes. The utilization of appropriate biopsy technique may lead to the diagnosis of classic KS tumors in populations outside of the current four widely accepted clinical subtypes.

## Background

Kaposi sarcoma (KS) is a vascular tumor caused by the proliferation of spindle cells in the endothelium of blood vessels [1]. In the United States there are four widely accepted distinct KS clinical subtypes. *Classic* KS, typically benign and mostly limited to the hands and the feet, is seen primarily in elderly men of Eastern European and Mediterranean origin [2]. *Endemic* or African KS is most commonly seen in indigenous Sub-Saharan Africans and is independent of the human immunodeficiency virus (HIV) [3]. *Iatrogenic* KS is associated with recipients of organ transplants and patients undergoing immunosuppression therapy [4]. *Epidemic* or acquired immune deficiency syndrome (AIDS) KS is the most common tumor seen in people infected with HIV. This subtype is most frequently described in HIV seropositive homosexual and bisexual men [2, 5]. Each KS clinical subtype is linked through infection of the Kaposi sarcoma-associated herpes virus (KSHV), also known as human herpesvirus 8 (HHV-8) [6]. We present a case describing the unusual presentation of classic KS in a diabetic African-American heterosexual HIV seronegative male.

## Case presentation

**Fig. 1:**
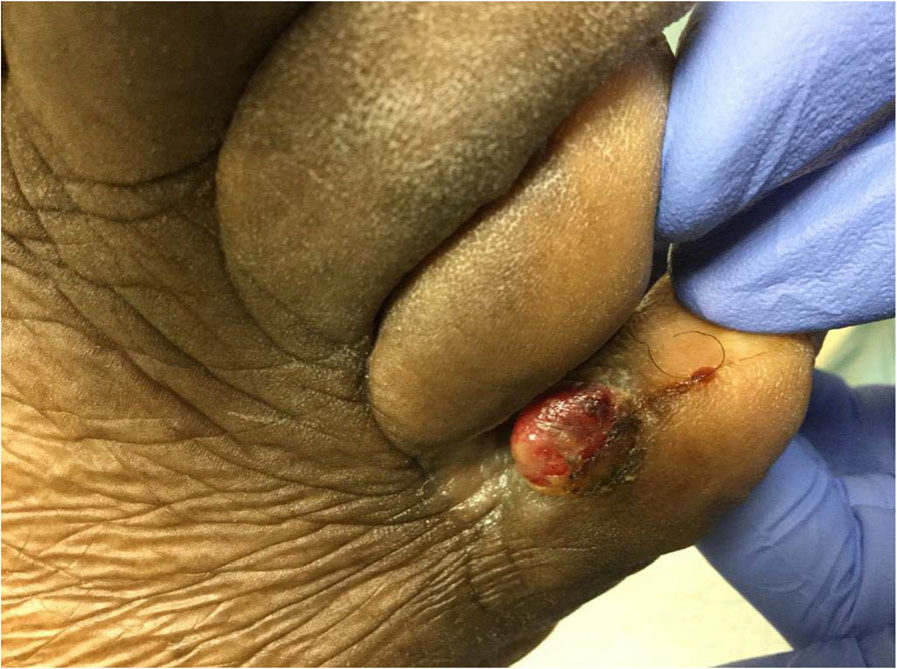
Right foot medial fifth digit with granulomatous mass

**Fig. 2:**
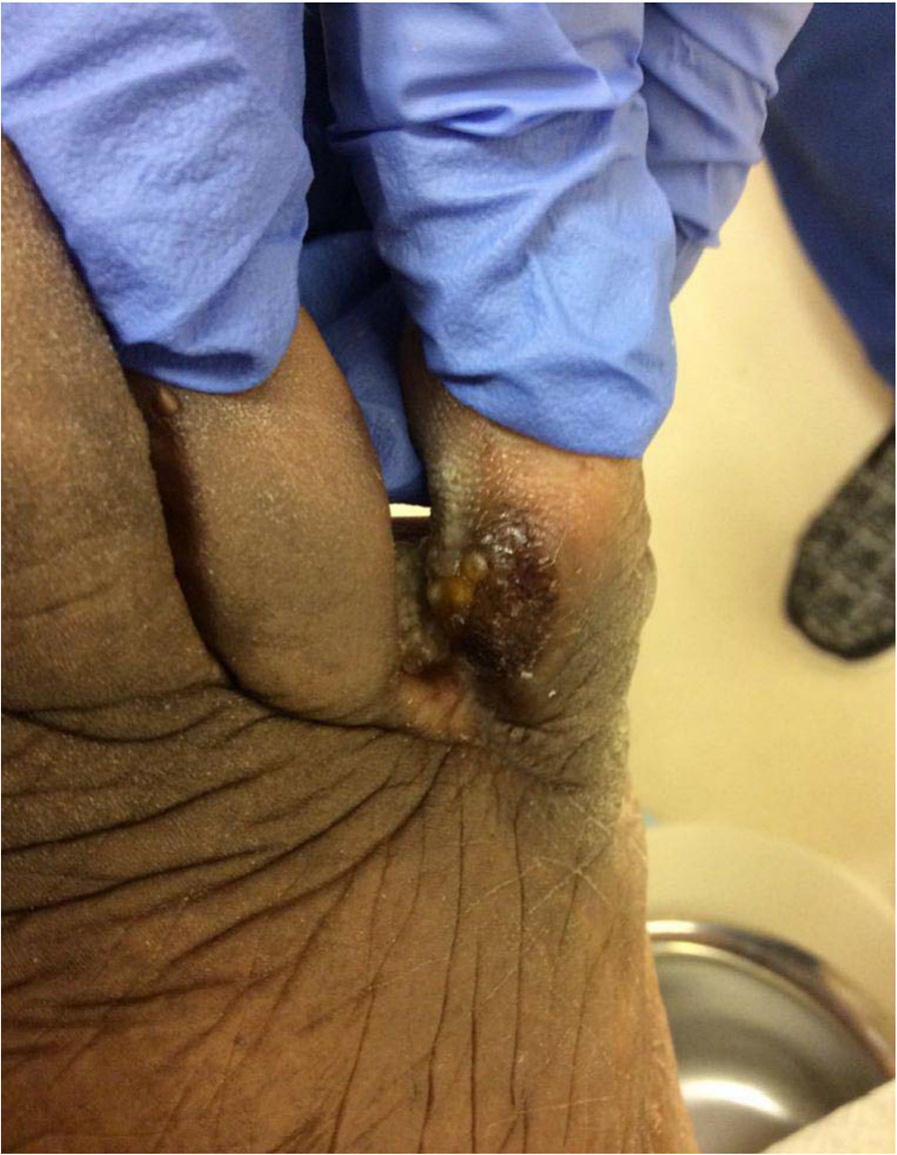
Right foot fifth digit partial thickness wound with sloughed granulomatous mass

An 80-year-old African-American male presented to the University of Michigan Hospital and Health Systems Comprehensive Wound Center for ongoing care of a painful right foot fifth digit wound. He first noticed the lesion after stubbing the digit 2 months prior. He presented to his primary care doctor 1 month after the injury and the lesion was diagnosed as a diabetic foot ulcer (DFU). The lesion improved minimally with local wound care. Due to stagnation of the suspected DFU the patient presented to the wound center for further care. Upon initial wound center presentation, a granulomatous mass was noted to the medial aspect of the right fifth digit (Fig. 1). A biopsy of the lesion was subsequently scheduled. However, upon presenting to the biopsy procedure, the patient stated the granulomatous mass had sloughed off in his sock the week prior leaving only a small partial-thickness ulcer (Fig. 2). The patient elected to defer the biopsy as his symptoms had improved. The patient continued regular monthly follow-up at the wound center with development of his wound into two painful papules.

**Fig. 3:**
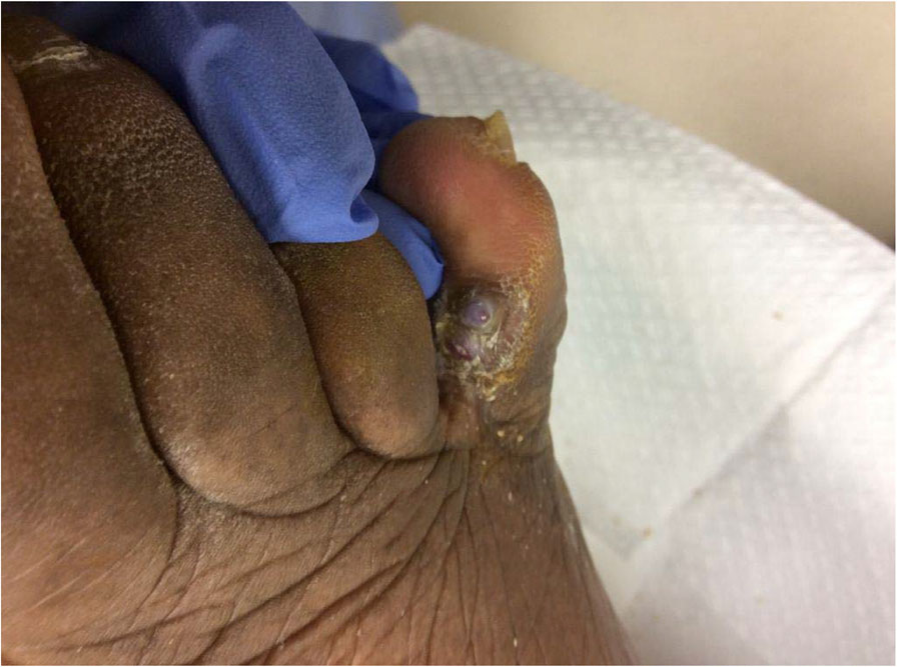
Right foot fifth digit with two distinct cyanotic papules

The patient’s past medical history was significant for type 2 diabetes mellitus T2DM, hypertension, hyperlipidemia, asthma, and dementia. He denied a family history of skin lesions and cancer. He did not take any immunosuppressive medication. The patient was a lifelong non-smoker and denied alcohol and illicit drug use. He was married, heterosexual and monogamous. At the time of presentation his last recorded hemoglobin A1c was 6.1%. Clinical examination revealed palpable pedal pulses with triphasic flow to the bilateral dorsalis pedis artery and posterior tibialis artery. His right hallux toe pressure was 126 mmHg and his left hallux toe pressure was 184 mmHg. He had diminished protective sensation, 2 out of 4 sites bilaterally, as tested with 10-g 5.07 Semmes-Weinstein monofilament as per American Diabetes Association guidelines [7]. To the medial aspect of the patient’s right fifth digit were two firm, cyanotic appearing papules. The distal papule measured 0.5 cm in diameter and the proximal papule measured 0.7 cm in diameter (Fig. 3). There were no clinical signs of infection noted to the right foot. As the lesion caused moderate discomfort and did not improve despite 2 months of local care at the wound center, a biopsy of the lesion was again recommended.

**Fig. 4:**
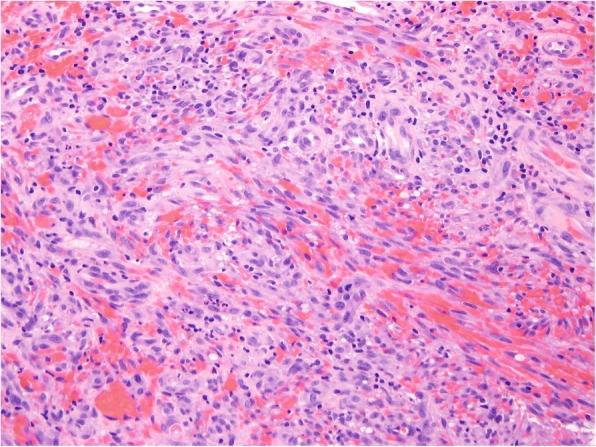
Histopathology hematoxylin and eosin stain with magnification 200X showing spindle cell proliferation and slit-like vascular spaces

**Fig. 5:**
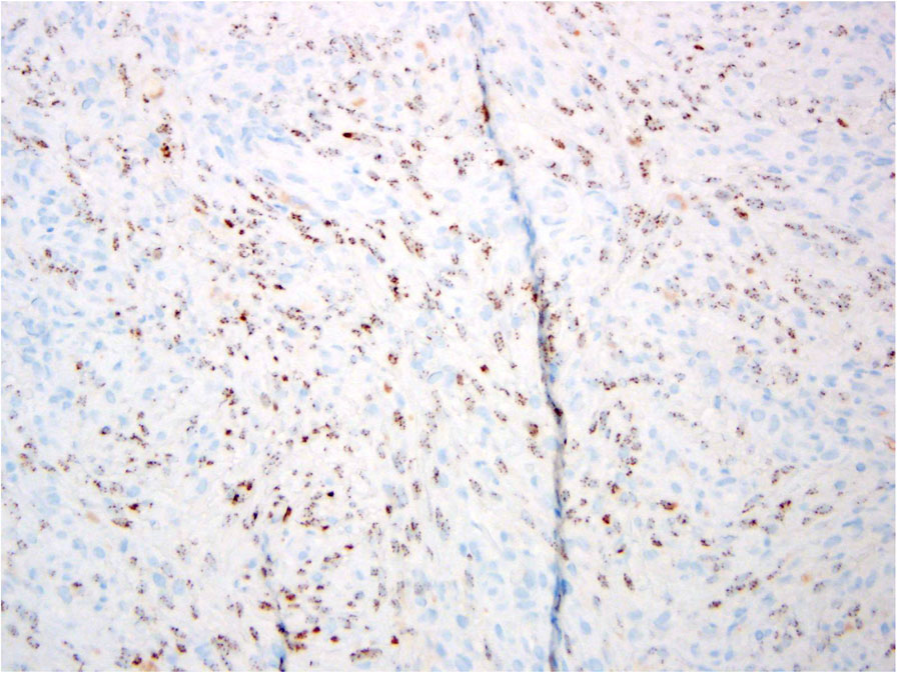
Proliferated spindle cells demonstrate positive staining (brown) of the antibody to HHV-8

After obtaining written informed consent a #15 blade was used to perform a shave biopsy of both the right fifth digit proximal and distal papules. The two specimens were labeled appropriately and sent separately to the University of Michigan Hospital and Health Systems pathology laboratory for assessment. The pathology report identified the specimen obtained from the distal papule as the superficial aspect of a hemorrhagic vascular lesion. The specimen from the proximal papule was identified as Kaposi sarcoma, nodular stage, transected at the deep margin. Hematoxylin and eosin stain demonstrated dermal nodules with haphazard spindle cell proliferation of endothelial cells forming vascular spaces which is diagnostic of classic KS (Fig. 4). The tumor cells showed diagnostic positive staining for HHV-8 (Fig. 5).

After the resulting classic KS diagnosis, the patient was referred to the Surgical Oncology Clinic at the University of Michigan Hospital and Health Systems. He underwent a series of blood tests to assess for underlying immunosuppression including HIV testing and a complete blood count with platelet and differential. He was determined to be HIV seronegative without any other clinical findings of immunosuppression. His case was reviewed by the University of Michigan Sarcoma Medical Oncology Department as well as the Multidisciplinary Sarcoma Tumor Board. The consensus of the combined board was local therapy with radiation without surgical intervention. The patient underwent 30 Gy of radiation in 10 fractions directed to the right foot fifth digit. The patient tolerated radiotherapy well and upon finishing his course of therapy, the KS lesion to the right foot had completely resolved. Upon a 10 month follow-up from initiation of radiation, the patient was disease free.

## Discussion

KS is rare in the general population. The disease represents about 1% of all diagnosed cancers worldwide [8]. The case we present is unusual as the clinical characteristics of the patient discussed do not fit those typical of classic KS or any of the other KS subtypes seen in the current literature. Classic KS is seen most commonly in men (male to female ratio 10–15:1) of Mediterranean or Eastern European origin. It is particularly seen in Ashkenazic Jews in their fifth through eighth decade of life [1, 4]. As classic KS most often affects the feet, it is not uncommon for KS lesions to be initially misdiagnosed as diabetic foot ulcers [9–11]. There are notable differences that can aid in distinguishing between DFUs and KS lesions. DFUs are typically divided into either neuropathic ulcers or neuroischemic ulcers [12]. Neuropathic ulcers, associated with diabetic peripheral neuropathy, are typically found on the weightbearing surfaces of a well-perfused foot. Common wound locations include the plantar metatarsal heads or the heel. Diabetic neuropathic ulcers are typically painless with well-defined wound margins. An important feature of neuropathic ulcers is peri-ulcer callus development. Callus formation is the result of repetitive mechanical stress, usually pressure and friction, in an insensate foot. If a callus is left intact for a prolonged period of time, the tissue underlying the callus will ulcerate. Neuroischemic ulcers have the underlying etiology of neuropathy and poor arterial perfusion. The foot type is usually pulseless, cool to touch, with atrophic skin and diminished pedal hair. Neuroischemic wounds commonly appear on the margins of the foot such as the foot edge, distal aspects of toes and back of the heel. The wounds can occur spontaneously or as a result of microtrauma [13]. Because of ischemia, neuroischemic wounds can be quite painful. A significant factor of DFUs is that once the underlying etiology is controlled, i.e. pressure or ischemia, the wound should improve or heal with local wound care.

Though the patient in our case study had peripheral neuropathy, classic KS lesions can occur in diabetic patients independent of neuropathy or ischemia. The lesions are usually on the soles and arches of the foot [14]. Pedal interdigital presentation of KS lesions is possible as well, which differs from the usual presentations of DFUs. Also, in contrast to DFUs, it is unusual for a classic KS lesion to initially present as a ulcer, with calloused tissue, or as a blister. KS lesions generally present as painless, violaceous plaques and nodules [15]. The lesions can appear either isolated or in groups. Enlargement and ulceration of a KS lesion occurs over prolonged periods of time [16]. Unlike DFUs, local wound care and removal of the suspected etiology will not result in resolution of the KS lesion as it is a tumor of viral origin.

The case presented serves as an example of the necessity to biopsy pedal lesions that do not respond to standard therapy. In addition to DFUs classic KS lesions can mimic other conditions such as pyogenic granuloma, melanoma, melanocytic nevi, arteriovenous malformations or severe stasis dermatitis [17]. The acronym “ABCDE” (asymmetry, border, color, diameter, and elevation) has longed served as a diagnostic protocol for melanoma. Additionally, ‘CUBED’ is an alternative acronym that can be used to aid clinicians in the diagnosis of suspicious lesions, particularly on the foot. In the ‘CUBED’ acronym: ‘C’ represents color of lesion; ‘U’ for uncertainty of diagnosis; ‘B’ describes bleeding lesions including chronic granulation tissue; “E” for enlargement or worsening of lesion despite local therapy; and ‘D’ describes delay in healing beyond 2 months [18]. If a pedal lesion displays any two features of the ‘CUBED’ acronym then biopsy is warranted.

In general patients with diabetes are more susceptible to pathogen infection and tumor growth [19, 20]. Several epidemiological studies describe a possible association between HHV-8 infection and T2DM [21]. More specific correlations have been described in recent literature. Infection with HHV-8 is needed for KS development. However, primary infection alone may not be sufficient for tumor development [6]. Once a patient is infected with HHV-8, the virus is typically latent. Ye et al. demonstrated that high blood glucose levels in patients with diabetes can promote HHV-8 lytic gene expression and replication leading to the development of classic KS [22]. Furthermore, Chang et al. showed that high glucose levels not only increase HHV-8 reactivation in latent cells, but also enhances the susceptibly of various host target cells to HHV-8 infection [23]. To date there is limited evidence related to a possible cause and effect nature of T2DM and HHV-8. More scientific investigations in this area can provide new understandings in diabetic complications and aid in risk stratifications.

The patient’s demographic designation is another interesting component of this case. As stated previously, classic KS is most commonly seen in elderly Mediterranean men. In recent years KS in African-American males has been linked to an HIV seropositive status [24]. African-American adults have a higher prevalence of T2DM than Whites [25]. Given the correlations of T2DM and KS, it may be possible that HIV seronegative African-Americans are at a higher risk of KS than previously thought. Classic KS is generally considered not life threatening [26]. Though, increased morbidity has been documented in African-Americans, who tend to have more diffuse tumors, lower cure rates, and decreased survival rates [15]. This could be associated with delayed diagnosis. Due to their violaceous hue, KS lesions can be difficult to detect in patients of darker complexion. Mora and Lee described seronegative HIV KS diagnosed histologically in 19 African-Americans from 1948 to 1983 [27]. Over 50 % of patients had KS lesions, specifically on the foot. Sixteen percent of patients had diabetes and the mortality rate of patients in this series was 21%. In the United States African Americans have the highest death rate and shortest survival rate for most cancers of any racial and ethnic group [28]. As such, clinicians should have a high degree of suspicion for malignancy for atypical skin lesions in this population.

The patient did report that his right fifth digit lesion was precipitated by stubbing the digit. Interestingly, pedal trauma induced KS has been discussed in the literature. Berkowitz et al. described a case in which a 48-year-old HIV positive man developed KS of the right hallux after dropping a piece of wood on the digit [29]. The authors attributed KS in this patient to Koebner phenomenon. Koebner phenomenon, also known as Koebner response, is the development of skin disease at a site of cutaneous injury in patients that are vulnerable to that disease. Though Koebner phenomenon is most commonly associated with psoriasis, it is accepted that KS can occasionally exhibit the response [30]. Even without immunodeficiency, several conditions, including infections, tumors and immune reactions, can locally appear on areas of the body that have been immunologically undermined by trauma [31].

In clinical practice punch biopsy is the most accepted biopsy technique for definitive diagnosis of a classic KS. Punch biopsy provides a full-thickness specimen, which allows for adequate depth for proper diagnosis [32, 33]. In this case report, a shave biopsy was used. The rationale for shave biopsy was the patient’s lesion was small (less than 1.0 cm) and relatively flat. Though in the case presented defining classic KS histologic characteristics were seen utilizing a shave biopsy technique, it must be stressed that superficial shave biopsies can certainly miss important diagnostic features located in deeper tissues. More extensive biopsy techniques include excisional, incisional and core needle. An excisional biopsy is an operative procedure that removes the entire lesion. Incisional biopsies remove only part of the lesion for diagnostic sampling and core needle involves using a fine needle to obtain cells and tissue from the lesion [34].

Treatment for KS depends on the type and number of lesions, the extent of the disease, as well as the overall patient health. [34]. There is no definitive cure for KS [35]. Radiation is now widely used and has been proven effective in treating patients with symptomatic local disease [1, 36]. Due to the local and symptomatic nature of the patient’s disease and his history of diabetes, our institutional Medical Oncology Department and Multidisciplinary Sarcoma Tumor Board recommended local therapy. In regards to radiation therapy there is no universally accepted dose and fractionation [35]. A higher cumulative dose tends to have better results than lower doses [36]. The patient presented was treated with 30 Gy of radiation in 10 fractions and had resolution of his disease. Alternative local disease treatment includes cryotherapy, topical retinoid treatment, curettage and electrodessication, photodynamic therapy and intralesional chemotherapy [34]. Patients with extensive disease or recurrent KS can be managed with a combination of surgery, radiation and chemotherapy [37].

## Conclusion

This case is atypical as it shows classic KS in a heterosexual, HIV-seronegative African-American male. Though KS is common in indigenous Africans, classic KS has been described as rare in the American black heterosexual and HIV-seronegative population. Growing literature suggests an association between diabetes and KS, though the specifics of the association are unclear. Given the current perceived rareness of KS in some populations, the clinician should be astute when examining suspicious pedal lesions in patients with diabetes to avoid misdiagnoses and delayed treatment.

## Data Availability

Data sharing not applicable to this article as no datasets were generated or analyzed during the current study.

## Abbreviations

AIDS: Acquired immune deficiency syndrome
DFU: Diabetic foot ulcer
T2DM: Type 2 diabetes mellitus
HHV-8: Human herpes virus 8
HIV: Human immunodeficiency virus
KS: Kaposi sarcoma
KSHV: Kaposi sarcoma-associated herpes virus
